# Ultrasonographic examination of the patellar ligament after capsular and fascial imbrication for the treatment of cranial cruciate ligament rupture in dogs

**DOI:** 10.3389/fvets.2025.1544445

**Published:** 2025-03-25

**Authors:** Mario Candela Andrade, Senta Maria Hoffmann, Pavel Slunsky, Ignacio De Rus Aznar, Leo Brunnberg

**Affiliations:** ^1^Department of Medicine, Health and Medical University, Potsdam, Germany; ^2^Tierarztpraxis am Tierheim Berlin, Berlin, Germany; ^3^AniCura Small Animal Clinic Augsburg, Augsburg, Germany; ^4^Shoulder Surgery Unit, Orthoapedic and Traumatology Department, CEMTRO Clinic, Madrid, Spain; ^5^Small Animal Clinic, Department of Veterinary Medicine, Freie Universität Berlin, Berlin, Germany

**Keywords:** knee, sonography, canine, anterior, surgery

## Abstract

**Introduction:**

Cranial cruciate ligament (CCL) rupture is the most common orthopedic condition in dogs, with many surgical options available for its treatment. Thickening of the patellar ligament after capsular and fascial imbrication (CFI) is a frequently reported complication, but its clinical significance remains unclear.

**Method:**

This prospective study evaluated patellar ligament structural and thickness changes after CCL repair using CFI. Forty-six dogs with CCL ruptures treated at the Small Animal Clinic of Freie Universität Berlin between July 2013 and April 2015 were included. Clinical, radiographic, and sonographic assessments were performed pre-surgery and at 2–3 months and 8–10 months post-surgery. Parameters assessed included lameness scores, joint effusion, stability, extension pain, muscular atrophy, and arthritis. Uninjured contralateral joints from 20 dogs served as controls.

**Results:**

Postoperative improvements included reduced lameness scores, joint effusion, instability, and extension pain. Patellar ligament thickness increased from 1.6 mm pre-surgery to 5.4 mm at 2–3 months, then decreased to 3.9 mm by 8–10 months. Structural changes peaked at the first follow-up (52.9%) and decreased by the second (6.4%). No significant correlations were found between ligament changes and clinical outcomes, patient demographics, or adjunct treatments like NSAIDs or physical therapy.

**Conclusion:**

Patellar ligament alterations following CFI appear temporary and largely unrelated to clinical signs, patient factors, or treatment variables. These findings suggest that such changes do not significantly impact postoperative outcomes, underscoring the reliability of CFI as a treatment option for CCL rupture in dogs.

## 1 Introduction

The cranial cruciate ligament (CCL) stabilizes the stifle joint by limiting hyperextension, internal rotation, and cranial movement of the tibia in relation to the femur (1) and its rupture is the most common cause of hindlimb lameness in dogs (2–4). Currently, there are multiple surgical techniques available, broadly categorized into intra-articular, extra-articular, and osteotomy techniques. Radiographically visible thickening of the patellar ligament is frequently described as a complication following Tibial Tuberosity Advancement (TTA) (5–8) and Tibial Plateau Leveling Osteotomy (TPLO) (9–12), and in one study, after capsular fascia imbrication (CFI) (13).

Although ultrasound is cost-effective, readily available, non-invasive, and significantly superior to X-rays for depicting tendon changes (14, 15), only three studies (5, 7, 11) have documented these findings using sonography. Furthermore, there is a lack of studies exploring the clinical significance of these changes after a CFI.

The aim of the present study is to evaluate the patellar ligament using ultrasound before and after CFI treatment of CCL rupture in dogs. This evaluation will include measuring structural changes in the ligament over time and correlating these changes with clinical and radiological parameters such as joint stability, range of motion, and osteoarthritis progression. Given that thickening of the patellar ligament has been observed with other surgical techniques for CCL rupture, this study seeks to investigate whether similar outcomes are associated with the CFI technique. By examining the relationship between the CFI technique and potential patellar ligament thickening, we aim to better understand the mechanical or biological factors that might contribute to this phenomenon.

Additionally, the study will explore the influence of variables such as age, gender, body weight, and preoperative, intraoperative, and postoperative conditions, including rehabilitation protocols. By considering these factors, the research intends to provide a comprehensive analysis of how they might interact with surgical outcomes and patellar ligament morphology.

## 2 Materials and methods

### 2.1 Patient group

This controlled prospective study included only dogs that were at least 1 year old. The animals were presented to the Small Animal Clinic of the Freie Universität Berlin, (Berlin, Germany) between July 2013 and April 2015 for partial or complete cranial cruciate ligament rupture (CCLR). The stifle joint was stabilized using a capsular fascia imbrication technique (CFI) (16). Dogs were excluded if they had another orthopedic condition besides cruciate ligament rupture, such as patellar luxation, osteochondritis dissecans, or a fracture in the same joint, or if they had previously undergone surgery on the same limb due to another condition. Dogs with chronic systemic endocrine diseases, such as hypothyroidism or Cushing's syndrome, were also excluded.

Each patient in the study underwent a general clinical and targeted orthopedic examination. A blood count and blood chemistry were conducted as additional components for assessing the dog's health. Additionally, thoracic X-rays were taken in the latero-lateral projection.

### 2.2 Clinical examination

During the general and specific orthopedic examination, the degree of lameness, joint effusion, and cranial drawer test were evaluated preoperatively, as well as 2–3 months and 8–12 months post-surgery, based on visual inspection at rest and in motion (Table 1) (17).

**Table 1:** Assessment criteria for lameness and stifle joint evaluation in dogs.

**Assessment category**	**Grade**	**Description**
Lameness degree	0	No detectable lameness, equal weight-bearing
	1	Mild, indistinct lameness, consistent use of the limb, with minimal observable offloading
	2	Clearly mild lameness, consistent use of the limb, with noticeable offloading
	3	Moderate lameness, limb is occasionally not used but still placed down
	4	Severe lameness, limb is not used
Joint effusion	0	No increased joint effusion, patellar ligament clearly defined
	1	Mild effusion, diffuse swelling palpable lateral and/or medial to the patellar ligament, but the tendon is distinguishable
	2	Moderate effusion, significant swelling palpable on both sides of the patellar ligament, tendon only palpable when the stifle joint is flexed
	3	Severe effusion, patellar ligament not palpable
Drawer sign	0	Stable, no drawer motion detectable
	1	Questionable instability, drawer motion up to 2 mm detectable
	2	Clear instability, drawer motion >2 mm detectable
Extension pain	0	No reaction to hyperextension of the stifle joint
	1	Mild pain response to hyperextension (tension, licking of the mouth)
	2	Clear pain response to hyperextension (avoidance behavior, vocalization)
Muscle atrophy	0	Musculature equally developed on both sides
	1	Slight muscle atrophy, difficult to detect by palpation
	2	Significant muscle atrophy, easily detected both palpably and visually

### 2.3 Radiological examination

The digital X-ray machine Optimus Bucky Diagnost TH2^®^ (Philips, Eindhoven, NL) was used to produce the X-rays, and the images were analyzed using a specialized imaging system (curaPACS^®^, cura systems, Karlsdorf, DE).

In the context of CCLR an X-ray of the pelvis was taken in the ventrodorsal projection, as well as lateral X-rays of both stifle joints in the mediolateral projection. This was done to document any changes such as hip dysplasia, coxarthrosis, gonarthrosis, and/or trochlear dysplasia, which could potentially influence the prognosis in relation to the upcoming cruciate ligament surgery. These findings could also serve as a reference for future follow-up evaluations by comparing the current status with the previous one.

In line with Brunnberg (17), any arthritic changes were assessed (Table 2). The individual points were summed to determine the degree of coxarthrosis and gonarthrosis/trochlear dysplasia. The evaluation included the acetabulum, joint space, femoral head, femoral neck and stifle joint.

**Table 2:** Grading criteria for osteoarthritis in hip and stifle joints—Brunnberg (17).

**Location**	**Grade**	**Description**
Hip joint	0	Acetabulum: deep, sharp contours, concentric joint space. Femoral Head: spherical, sharply contoured, deeply seated.
	1	Acetabulum: mild flattening, unclear contours, early deposits, slight joint space divergence. Femoral head: slightly rounded or unclear contours.
	2	Acetabulum: marked flattening, unclear contours, visible deposits, divergent joint space. Femoral Head: mushroom-shaped or angular.
	3	Acetabulum: significantly flattened, unclear, with prominent deposits and joint space divergence. Femoral Head: flattened, mushroom-shaped, or subluxated.
Stifle joint	0	Tibial condyles: sharp contours, distinct eminence, only posterior sulcus boundary visible. Femoral Condyles and Patella: sharp, smooth patella.
	1	Tibial condyles: unclear contours, flattened eminence, anterior and posterior sulcus boundaries visible. Femoral condyles and patella: mild blurring or slightly irregular patellar shape.
	2	Tibial condyles: clear erosion, deposit buildup. Femoral condyles and patella: visible irregularities, deposits on lateral or medial condyles.
	3	Tibial condyles: flattened eminence, extensive deposits. Femoral condyles and patella: prominent irregularities, extensive deposits, flattened contours.

The length of the patellar ligament was measured using the curaPACS^®^ software (cura systems, Karlsdorf, DE) from the distal origin at the base of the patella to the proximal insertion at the tibial tuberosity.

### 2.4 Sonographic examination

**Figure 1:**
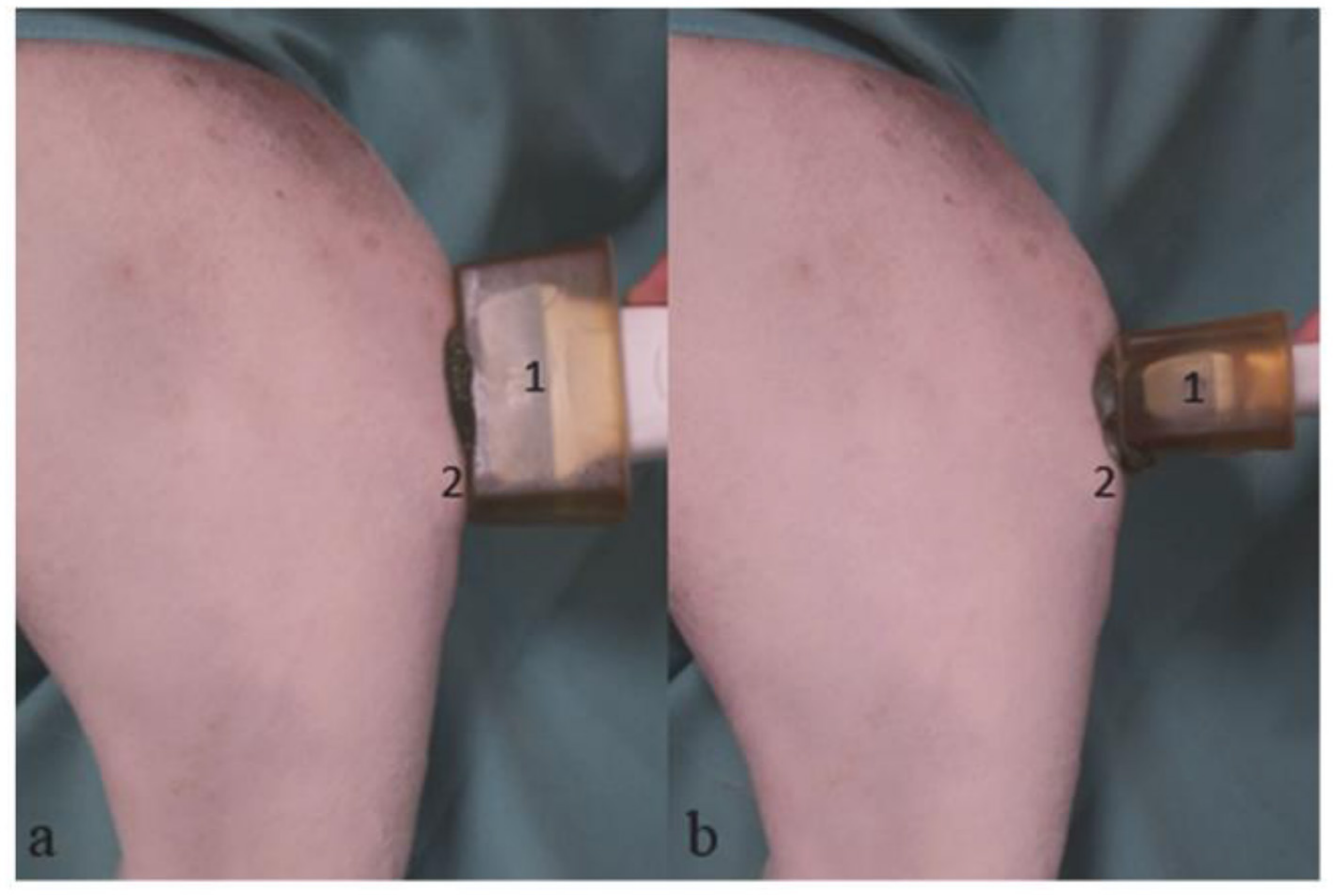
Mixed breed, 4 years old, male; sonographic examination of the right stifle joint. Sagittal section **(a)**, Transverse section **(b)**. (1) Linear transducer with lead-in distance, (2) Tibial tuberosity.

The stifle joints were examined sonographically under sedation or anesthesia for the initial pre-surgical examination, with the limb already shaved. Subsequent examinations did not require sedation. The other stifle joint was also shaved and sonographically examined for comparison. The shaving was limited to the smallest possible area while still allowing for adequate visualization (Figure 1).

During the examination, the animal was positioned laterally with the limb to be examined on top, held at a stifle joint angle of 135°. A linear transducer (11 MHz) from the Logiq P6^®^ machine (GE Healthcare, Chalfont St. Giles, UK) was applied cranially at a 90° angle to the patellar ligament (Figure 1). To better visualize the patellar ligament in smaller dogs, we used a spacer as recommended (14).

**Figure 2:**
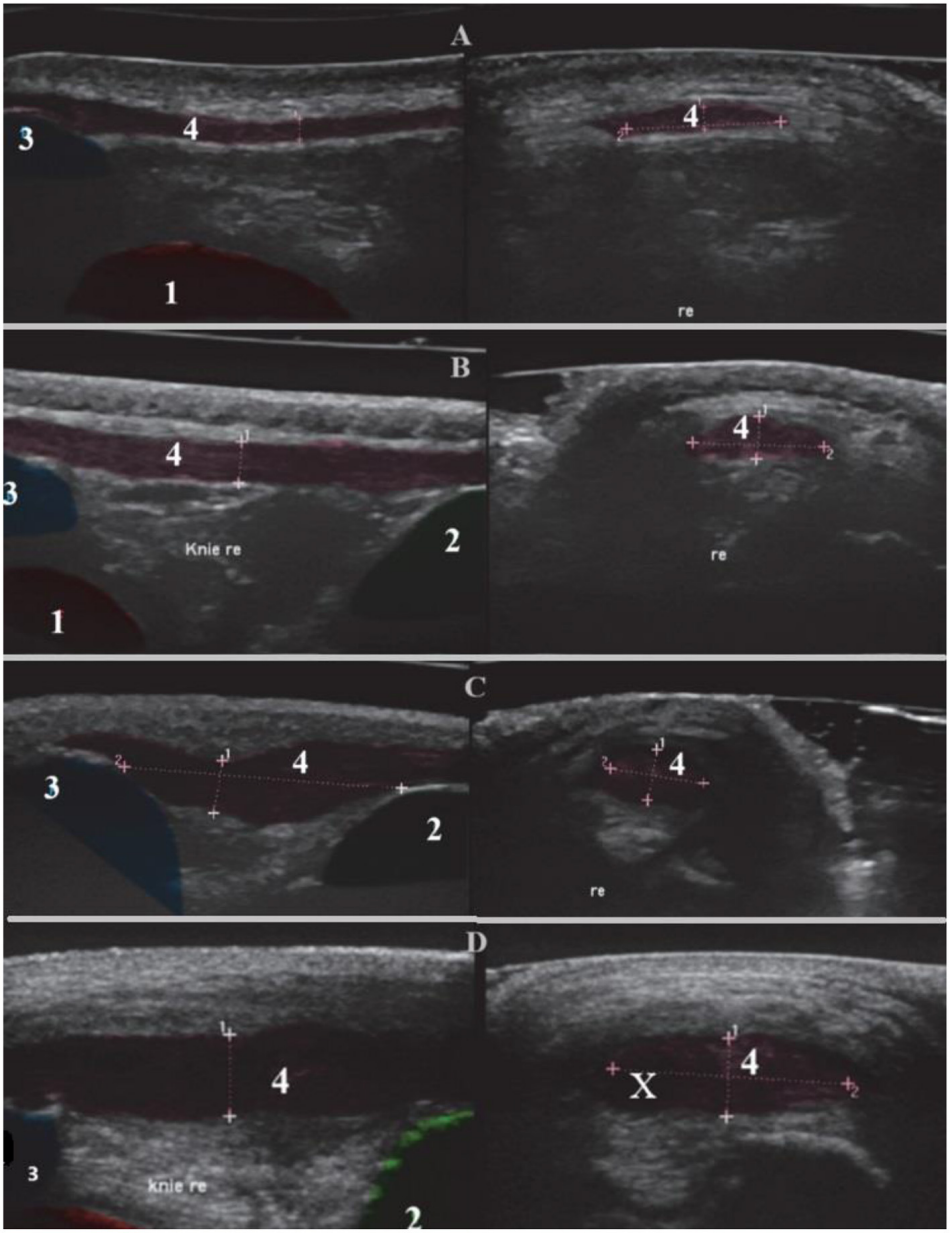
Sonographic representation of the right patellar ligament with changes. Sagittal section (left), transverse section (right). (1) Femur, (2) Tibia, (3) Patella, (4) Patellar ligament with fiber alignment, *Re* right and (X) Core lesion. **(A)** Grade 0, Boxer, 8 years old, female. **(B)** Grade I, Golden Retriever, 6 years old, female. **(C)** Grade II, German Shepherd, 9 years old, male. **(D)** Grade III, Mixed breed, 5 years old, male.

The thickness was measured at the midpoint of the ligament in cross-section. At each time point—pre-operatively (QI1), 2–3 months post-surgery (QI2), and 8–12 months post-surgery (QI3) the measurement was taken three times, and the mean value was calculated for statistical analysis. To obtain a size-independent variable, the quotient (QI) of tendon length and caliber was formed. Additionally, the contour, echogenicity, and periligamentous region were sonographically imaged and graded in both sagittal and cross-sectional views and graded according to Mattern et al. (11):

Grade 0 = Normal tendon thickness, homogeneous normal echogenicity, uniform fiber alignment, smooth contour; peritendinous structures unremarkable (Figure 2A).

Grade 1 = Tendon thickening with normal echogenicity, normal fiber alignment, and smooth contour; peritendinous structures unremarkable (Figure 2B).

Grade 2 = Tendon thickening with altered echogenicity, irregular fiber alignment, and contour; peritendinous hyperechogenicity (Figure 2C).

Grade 3 = Tendon thickening with pronounced anechoic areas (core lesions) and interruptions in fiber alignment; peritendinous hyperechogenicity or anechogenicity (edema) (Figure 2D).

### 2.5 Data collection

I. The signalment of the patients, including breed, age, sex, and body weight, was obtained from the medical records.
II. The duration of lameness was inquired and categorized as acute (<2 weeks) or chronic (lasting longer than 2 weeks).
III. If the patient had been pretreated with NSAIDs, the duration of administration was classified as short (within 4 weeks) or long (longer than 4 weeks).
IV. Intraoperatively, the status of the cruciate ligament tear (complete or partial) and the integrity of the medial meniscus were assessed, along with the extent of synovitis, capsular fibrosis, and gonarthritic/gonotrochlotic changes, as follows:
  - Mild: Slightly increased but otherwise unchanged synovia, no capsular fibrosis, no visible arthritic changes.
  - Moderate: Increased synovia with slight turbidity and/or liquefaction, capsular thickening, dull cartilage surface, and early bone formation.
  - Severe: Markedly increased, fluid synovia with significant turbidity or hemarthrosis, pronounced capsular fibrosis, significant cartilage damage (ulcerations), and prominent marginal protrusions.
V. At follow-up appointments, the duration of other medications, such as metamizole, NSAID usage, postoperative complications, physical therapy management, and any occurrence of contralateral cruciate ligament rupture were recorded.

### 2.6 Surgical procedure

All patients in the study were operated on using the modified capsular-fascial imbrication (CFI) technique described by Meutstege (16), performed by the same certified small animal surgery specialist.

#### 2.6.1 Premedication and anesthesia

An intravenous catheter (Braunüle^®^, B. Braun, Melsungen, Germany) was placed in the cephalic vein of either the right or left forelimb, and a single perioperative dose of 12.5 mg/kg amoxicillin-clavulanic acid (AmoxClav^®^, Hexal, Holzkirchen, Germany) was administered intravenously. For premedication, 0.25 mg/kg levomethadone (L-Polamivet^®^, Intervet, Unterschleißheim, Germany) and midazolam (0.1 mg/kg i.v., Midazolam^®^ B. Braun Melsungen AG, Germany) was injected intravenously. Anesthesia was induced with 2–6 mg/kg propofol (Narcofol^®^, CP-Pharma, Burgdorf, Germany) intravenously, allowing the animal to be intubated. Following sonographic examination of the stifle joints, inhalation anesthesia was initiated using a mixture of 1.5% isoflurane (IsoFlo^®^, Zoetis, Kalamazoo, UK) and oxygen. To compensate for perioperative and intraoperative fluid loss, a balanced electrolyte solution (Sterofundin^®^, B. Braun, Melsungen, Germany) was infused at a rate of 5–10 ml/kg/h intravenously. Continuous monitoring of blood pressure, pulse, ECG, respiration rate, and partial pressures of oxygen and carbon dioxide was performed throughout the anesthesia.

#### 2.6.2 Preparation

The limb to be operated on was shaved from the proximal third of the thigh to the tarsus, and this area was washed with a disinfecting soap (Braunoderm^®^, B. Braun, Melsungen, DE). The patient was placed in dorsal recumbency on the operating table in the surgery room. The limb was not fixed, allowing for manipulation of the stifle joint during the procedure (flexion, extension, abduction, adduction, internal and external rotation). The shaved area was disinfected with a skin disinfectant (Braunol^®^, B. Braun, Melsungen, DE), covered with sterile self-adhesive drapes, and the patient was draped with a large sterile disposable cloth.

#### 2.6.3 Surgery

The procedure followed the approach described by Allgöwer et al. (16). The patient was positioned in dorsal recumbency, and a lateral parapatellar approach to the stifle joint was used. After medially displacing the patella, a cleanup was performed, including the removal of remnants of the cranial cruciate ligament and, in cases of meniscal lesions, a partial medial meniscectomy. Two vertical mattress sutures were placed from the lateral fabellopatellar fiber cartilage to the insertion site of the patellar ligament. Depending on the weight class, USP 0 sutures were used for dogs <20 kg, USP 1 for dogs between 20 and 40 kg, and USP 2 for dogs >40 kg. A negative cranial drawer test confirmed sufficient stabilization. The joint capsule was closed with single interrupted sutures, and the fascia doubling was completed using horizontal mattress sutures with the same suture material. The subcutaneous tissue was closed with simple interrupted sutures (Monocryl^®^, Johnson & Johnson, Diegem, BE), and the skin was closed with Sultan's diagonal sutures (Ethilon^®^, Johnson & Johnson, Diegem, BE).

#### 2.6.4 Postoperative care

After the operation, the stifle joint was immobilized for one day in a padded, protective Robert Jones bandage reinforced with crepe paper. For postoperative analgesia, Metamizol (Novaminsulfon^®^, Ratiopharm, Ulm, Germany) was prescribed for 3–7 days at a dose of 20–30 mg/kg, administered 2–3 times daily orally, as well as other NSAIDs like carprofen (Rimadyl^®^, Zoetis, Karlsruhe, Germany) (2 mg/kg q24h), cimicoxib [Cimalgex (Cimicoxib, Vetoquinol, Lyon, France) (2mg/kg q24h), or firocoxib (Previcox^®^, Merial, Lyon, France) (5mg/kg q24h)]. The skin sutures were removed 10–14 days post-operation. Owners were advised to follow the following movement management plan:

- For 2 weeks, a maximum of 10 min walks, 3–4 times daily on a leash.
- Increase walk duration by 10 min each week over a total of 8 weeks.
- Begin physiotherapy when possible after the removal of skin sutures.

### 2.7 Statistical analysis

Data were analyzed using IBM SPSS Statistics^®^ software (Armonk, United States), Version 23.0 for Windows. Changes in tendon thickness and ordinal variables over time were assessed using the Wilcoxon signed-rank test for comparisons between individual time points and Friedman's two-way analysis of variance for all three time periods combined. The Wilcoxon signed-rank test was also used to compare the ratios of the operated and contralateral limbs. The healthy contralateral stifle joints were used as a control group. Potential associations between anamnesis and intraoperative categories were analyzed using cross-tabulations and illustrated in corresponding diagrams. Trends were examined with the Chi-square test and Fisher's exact test for independence.

The groups were coded alphabetically with lowercase letters (a, b, c) to represent any statistical significance. The key derived from the numeric and alphabetic code is as follows:

1 = 2 = 3 → a, a, a

(no significant difference between 1, 2, and 3).

1 ≠ 2 ≠ 3 → a, b, c

(significant difference between 1, 2, and 3).

1 ≠ 2 + 1 ≠ 3 + 2 = 3 → a, b, b

(significant difference between 1 and 2, and 1 and 3, but no significant difference between 2 and 3).

Categorical and ordinal variables that could influence the quotient QI pre-operative, 2–3 or 8–10 months post-operatively were tested with the Kruskal-Wallis test (for more than two subgroups) or the Mann-Whitney U-test (for two-group comparisons) for unpaired samples. Correlations between tendon thickness and clinical parameters were analyzed using Kendall's Tau-b correlation coefficient. To evaluate the accuracy of sonographic measurements over time, the coefficient of variation for the QI of the control group was calculated. The significance level for the tests was set at 0.01.

## 3 Results

### 3.1 Patient population

#### 3.1.1 Epidemiology

A total of 46 dogs with 49 affected stifle joints were included in the study. Mixed-breed dogs (*n* = 20, 43.5%) of various types were the most commonly affected by cranial cruciate ligament rupture (CCLR), followed by Rottweilers (*n* = 4, 8.7%), Labrador Retrievers (*n* = 3, 6.5%), Boxers (*n* = 3, 6.5%), German Shepherds (*n* = 2, 4.3%), and Golden Retrievers (*n* = 2, 4.3%). The remaining breeds, including Staffordshire Bull Terrier, English Bulldog, Fox Terrier, French Bulldog, Pyrenean Shepherd, Jack Russell Terrier, Standard Poodle, Bernese Mountain Dog, Hungarian Vizsla, Dalmatian, Polish Lowland Sheepdog, and Great Dane, were each represented by one patient (2.2%).

Of the patients, 24 (52.2%) were male and 22 (47.8%) were female. Among the males, 15 (65.5%) were neutered, while 10 (45.5%) of the females were spayed.

The dogs were aged between 1 and 14 years (average 7.2) and weighed between 8 and 62 kg (average 29.5). The average age and weight related to the breeds are presented in Table 3.

**Table 3:** Breed distribution, average age, and weight of the dogs in the study sample.

**Breed**	**Number (*n*)**	**Percentage (%)**	**Average age (years)**	**Average weight (kg)**
Mixed Breed	20	43.5	7.6	24.5
Rottweiler	4	8.7	6.5	47.5
Labrador Retriever	3	6.5	8.5	33.3
Boxer	3	6.5	11	35.3
Golden Retriever	2	4.3	4	30
German Shepherd	2	4.3	6	45
Staffordshire Bull Terrier	1	2.2	7	21.5
English Bulldog	1	2.2	2	32
Fox Terrier	1	2.2	10	8
French Bulldog	1	2.2	5	13
Pyrenean Shepherd	1	2.2	8	10
Jack Russell Terrier	1	2.2	13	8
King Poodle	1	2.2	6	29
Bernese Mountain Dog	1	2.2	7	50
Hungarian Vizsla	1	2.2	5	33
Dalmatian	1	2.2	11	30
Pony	1	2.2	8	27
Great Dane	1	2.2	10	60
Total	46	100.0		

#### 3.1.2 Rupture of the cranial cruciate ligament

The cranial cruciate ligament was ruptured in 22 patients on the left side (47.8%) and in 21 patients on the right side (45.7%). In 3 animals, the ligament ruptured contralaterally during the examination period. According to the history, 10 (21.7%) dogs had previously experienced a contralateral cruciate ligament rupture and had undergone surgical treatment elsewhere. The specific surgical methods could not be precisely identified. No damage to the caudal cruciate ligament was diagnosed. There was acute lameness (≤2 weeks) in 19 (38.8%) cases, while 30 (61.2%) patients had been lame for more than 2 weeks.

#### 3.1.3 Pre-surgical treatment

Among ligament ruptures, 55.1% (*n* = 27) were pre-treated with NSAIDs for up to 4 weeks prior to surgery, 10.2% (*n* = 5) had their last NSAID application more than 4 weeks prior, and 34.7% (*n* = 17) received no NSAIDs. NSAID use was more frequent in cases of prolonged lameness (60%) than in cases of acute lameness (40%) before surgery, as shown in Supplementary Figure 1; however, this difference was not statistically significant (*p* = 0.121).

#### 3.1.4 Intraoperative findings

The cranial cruciate ligament (CCL) was fully ruptured in 30 (61.2%) stifle joints and partially ruptured in 19 (38.8%). The caudal horn of the medial meniscus was damaged in 33 (67.3%) cases, requiring partial resection. As shown in the descriptive comparison (Supplementary Figure 2), the proportion of meniscal damage was higher in animals with a complete ligament rupture (77.5%) than in those with a partial rupture (52.5%), though this difference was not statistically significant (*p* = 0.116). The distributions of patients with either a complete or partial rupture and with intact or damaged menisci were similar between animals with acute and chronic lameness.

The extent of other intraoperative findings such as synovitis, capsular fibrosis, gonotrochlosis, and/or gonarthrosis was mild in 22 (44.9%) joints, moderate in 19 (38.8%), and severe in 8 (16.3%).

**Figure 3:**
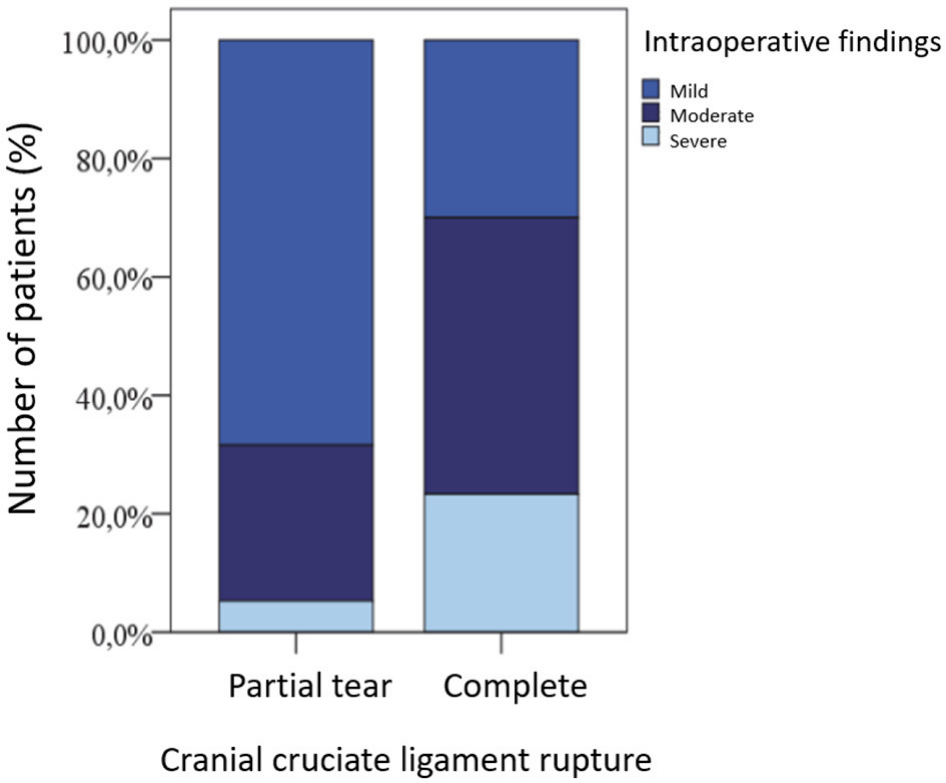
Extent of stifle joint changes in dogs with partial vs. complete cranial cruciate ligament rupture.

Patients with a complete rupture of the cranial cruciate ligament (LCC) showed more severe intraoperative changes than those with a partial rupture. This is visualized in Figure 3, although the difference was not statistically significant (*p* = 0.483).

#### 3.1.5 Postoperative course

In 28 cases (75.7%), metamizol was administered for <2 weeks, while in 9 cases (24.3%), it was given for a longer duration. Due to persistent lameness and significant joint swelling, 8 patients (21.6%) received additional NSAIDs, such as carprofen, cimicoxib, or firocoxib.

Complications occurred in 6 (16.2%) CCL surgeries, consisting of 5 infections and 1 suture reaction. The patients were treated with limb immobilization, and antibiotics. One case required surgical intervention. This dog was later euthanized due to a lung adenocarcinoma, excluding it from further follow-ups. Physiotherapy was performed in 11 (29.7%) cases.

### 3.2 Clinical examination

Out of 49 stifle joints in 46 dogs, 37 (75.5%) were examined at the first follow-up and 34 (69.4%) at the second follow-up. For the statistical comparison of clinical, radiological, and sonographic findings over the respective periods, only the initially affected joint was analyzed in the three dogs with bilateral ruptures to exclude any dependencies. The results of the clinical examination will be discussed in the following sections and presented graphically.

#### 3.2.1 Lameness grade

**Figure 4:**
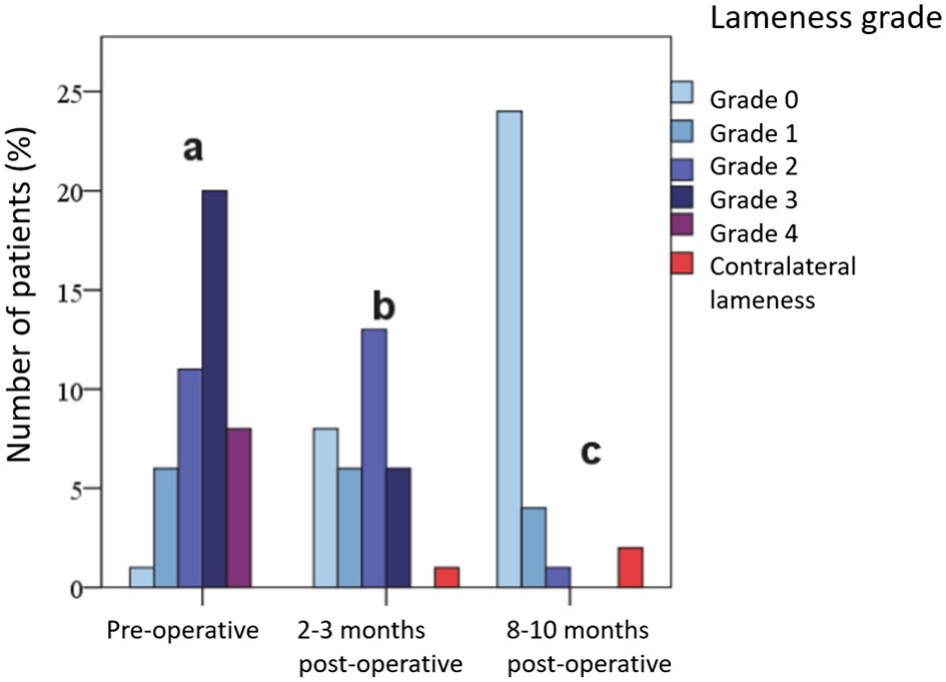
Results of lameness examination before and at various time points after surgery.

Prior to surgery, 60.9% (*n* = 28) of patients showed moderate to severe lameness (Grades 3 and 4). One patient was free from lameness, and 37.0% (*n* = 17) showed mild lameness (Grades 1 and 2). At the first follow-up (2–3 months post-operation), no dog showed severe lameness, and 9 (26.5%) patients were free from lameness. In total, 79.4% (*n* = 27) had only a mild grade of lameness at most (Grades 0, 1, and 2). One Lameness due to a contralateral cruciate ligament rupture was also observed. At the second follow-up (8–10 months post-operation), 24 (77.4%) dogs were free from lameness, and none showed moderate or severe lameness (Grades 3 and 4). However, two had contralateral lameness due to a cruciate ligament rupture. The differences in lameness severity between the different examination times were statistically significant (Figure 4).

#### 3.2.2 Joint effusion

Initially, all patients had joint effusion on the ruptured side, with 76.1% (*n* = 35) showing moderate to severe effusion (Grades 2 and 3) and 23.9% (*n* = 11) showing mild effusion (Grade 1). Post-operatively, the degree of effusion decreased significantly compared to preoperative levels. At 2–3 and 8–10 months post-operation, 52.9% (*n* = 18) and 67.7% (*n* = 21) of joints showed only mild or minimal effusion, respectively (Supplementary Figure 5). However, these differences were not statistically significant. Even after 8–10 months, severe joint effusion remained palpable in 2 cases (6.5%). Capsular fibrosis was palpable in all stifle joints post-operatively.

#### 3.2.3 Cranial drawer test

In 84.8% (*n* = 39) of patients, a distinct drawer sign was detectable on the rupture side before surgery, while only three joints (6.5%), those with partial ruptures seen during arthrotomy, were stable. At the 2–3 month follow-up, 41.2% (*n* = 14) of stifle joints remained distinctly unstable, whereas 35.3% (*n* = 12) had regained stability. By the 8–10 month follow-up, 22.6% (*n* = 7) of joints remained unstable, while stability was observed in 54.8% (*n* = 17). Significant differences were found between pre- and post-operative outcomes (*p* < 0.05), but not between the two follow-up periods (Supplementary Figure 6).

#### 3.2.4 Hyperextension pain

In the hyperextension test of the affected stifle joint, every animal reacted with pain before surgery, with 93.5% (*n* = 43) showing significant pain and 6.5% (*n* = 3) showing mild pain. This percentage of significant pain dropped to 26.5% (*n* = 9) at 2–3 months post-operation, with 35.3% (*n* = 12) showing mild pain and 32.4% (*n* = 11) showing no pain. At the 8–12 month follow-up, 12.9% (*n* = 4) of animals still exhibited significant pain, 25.8% (*n* = 8) had mild pain, and 61.3% (*n* = 19) had no pain. There was a significant difference between the pre- and post-operative results (*p* < 0.01), but not between the two post-operative examinations.

#### 3.2.5 Muscle atrophy

At the initial examination, 45.7% (*n* = 21) of patients had no muscle atrophy in the affected limb before surgery. Among the 8 animals (17.4%) with significant pre-operative muscle atrophy, 62.5% (*n* = 5) had been limping for over two weeks. At 2–3 months post-operation, 35.3% (*n* = 12) showed mild atrophy, 52.9% (*n* = 18) exhibited strong atrophy, and 11.8% (*n* = 4) showed no atrophy. By 8–12 months post-operation, 38.7% (*n* = 12) of patients had no palpable difference between hind limbs, 54.8% (*n* = 17) showed mild muscle atrophy, and only 6.5% (*n* = 2) had significant atrophy remaining. The distribution differences between the first and second, as well as between the second and third examination periods, were each significant (*p* < 0.01). However, there was no significant difference between the first and third time points.

#### 3.2.6 Radiological examination

The radiological findings regarding arthropathia deformans of the ipsilateral and contralateral hip joints are summarized in Table 4. There was no significant difference in the severity of osteoarthritis between both sides. No associations were found between the degree of lameness, gonarthrosis, or ligamentous changes.

**Table 4:** Severity of coxarthrosis ipsilateral/contralateral to the cruciate ligament rupture side.

**Classification**	**Ipsilateral**	**Percent (%)**	**Contralateral**	**Percent (%)**
Free—Grade 0	12	26.1	10	21.7
Mild—Grade I	15	32.6	17	37.0
Moderate—Grade II	14	30.4	13	28.3
Severe—Grade III	2	4.3	3	6.5
Total	46	100.0	46	100.0

**Figure 5:**
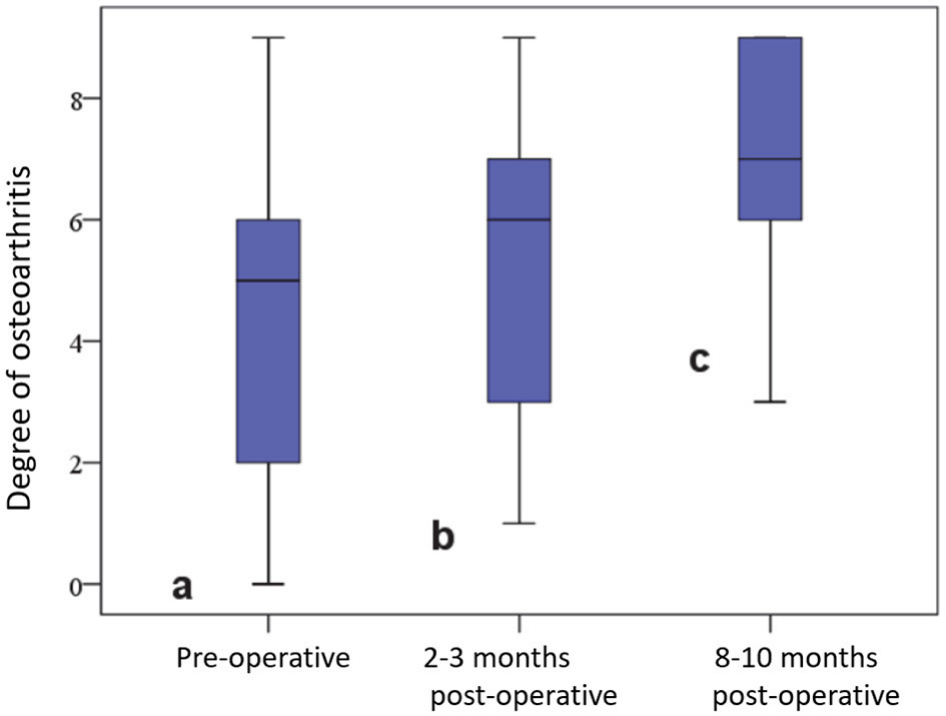
Degree of osteoarthritis in the affected stifle joint before and at various time points after surgery.

At the beginning, a capsular shadow was radiologically detectable in 47 (95.9%) joints, and an anterior drawer position was detectable in 5 (10.2%) joints. The degree of osteoarthritis in the affected stifle joint was 4.61 ± 2.63 pre-operatively, 5.65 ± 2.46 at 2–3 months post-operation, and 7.13 ± 1.73 after 8–10 months. These differences were significant (*p* < 0.01) (Figure 5).

The radiological measurement of the patellar ligament revealed a length of 3.67 ± 0.99 cm pre-operatively, 3.62 ± 1.04 cm at the first post-operative examination, and 3.57 ± 1.07 cm at the second.

#### 3.2.7 Sonographic examination

**Figure 6:**
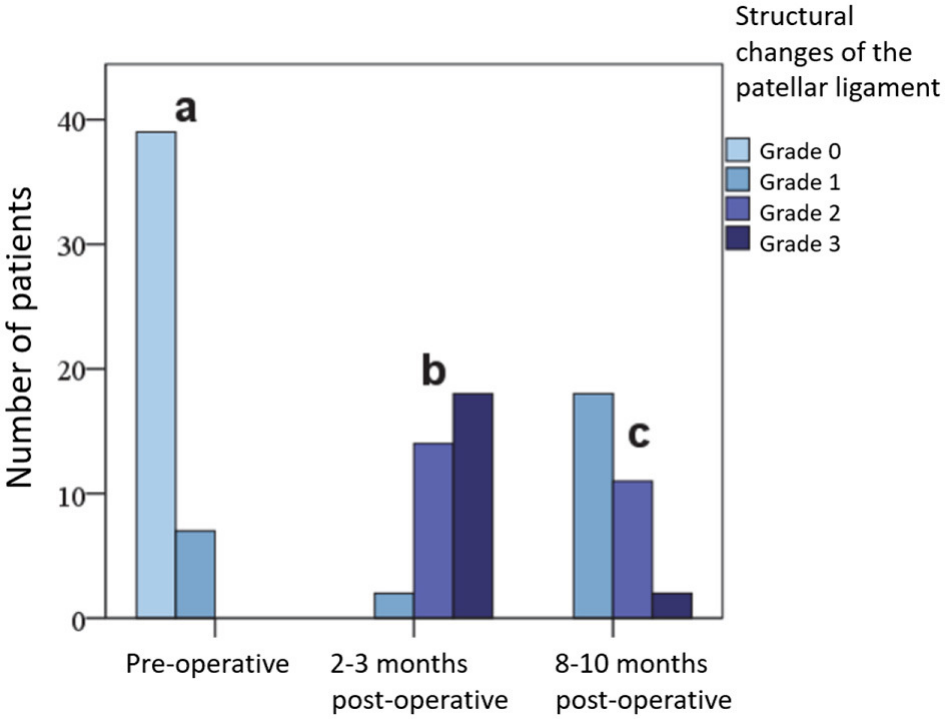
Extent of changes in the patellar ligament in grades (0, 1, 2, 3) on sonography before and at various time points after surgery.

Initially, the patellar ligament was sonographically assessed in 39 (84.8%) stifle joints as having no pathological findings and in 7 (15.2%) as mildly (Grade 1) altered. At 2–3 months post-operation, it was structurally moderately (Grade 2) altered in 14 (41.2%) cases and severely (Grade 3) altered in 18 (52.9%) cases, while at the 8–10 month post-operative examination, 18 (58.1%) tendons were still mildly (Grade 1) altered and only 2 (6.4%) were severely (Grade 3) altered. No tendon showed an identical structure when comparing sides for control. The differences were significant at all examination times and are presented in the form of bar charts in Figure 6.

The tendon caliber averaged 0.16 (0.09–0.26) cm pre-operatively on the ligament rupture side, 0.54 (0.22–1.08) cm at 2–3 months post-operation, and 0.39 (0.16–0.92) cm after 8–10 months (Supplementary Figure 4). In the healthy stifle joint, it averaged 0.15 cm at all examination time points.

**Figure 7:**
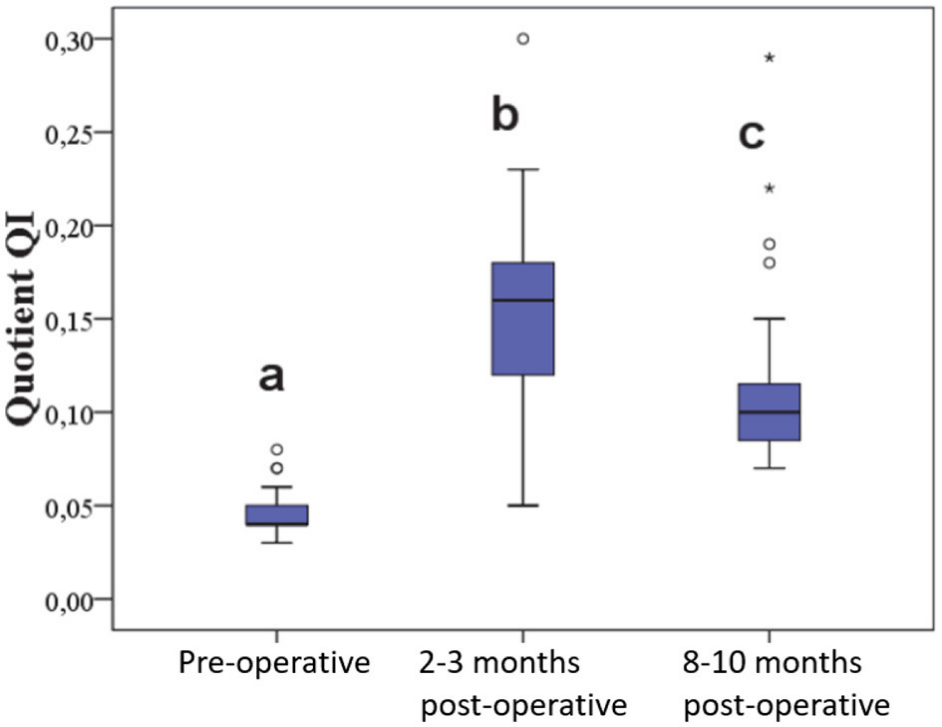
Quotient of length and caliber of the patellar ligament before and at various time points after surgery.

The quotient QI in the affected stifle joint was 0.045 ± 0.01 at the beginning, increased to 0.157 ± 0.05 at the first follow-up, and decreased to 0.11 ± 0.05 at the second follow-up. This was statistically significant (Figure 7).

In the uninjured contralateral stifle joints (*n* = 20), no significant differences were observed between the pre-operative and the two post-operative measurements. The coefficient of variation for QI in the control group was 11%.

### 3.3 Other correlations

#### 3.3.1 Age and weight for CCLR

**Figure 8:**
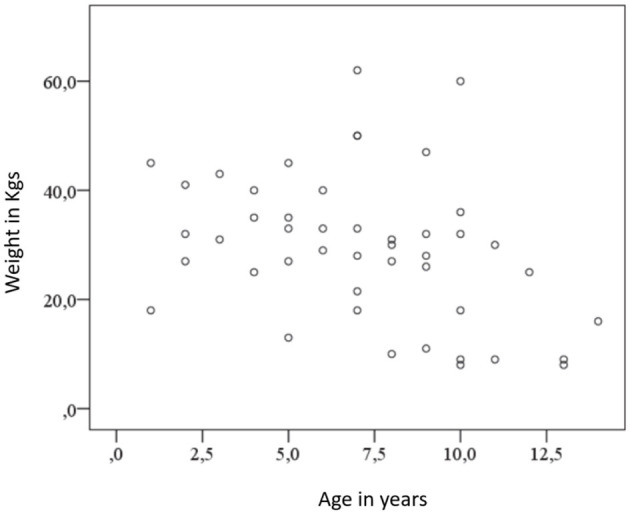
Negative correlation between age and weight.

When analyzing the distribution of age and weight, it was noted that smaller or lighter dogs were more likely to be presented at an older age, while heavier dogs were presented at a younger age. As shown in Figure 8, there is a statistically weak negative correlation between age and weight (*r* = −0.293, *p* = 0.006).

#### 3.3.2 Tendon length and caliber pre-operative (QI) and body weight

**Figure 9:**
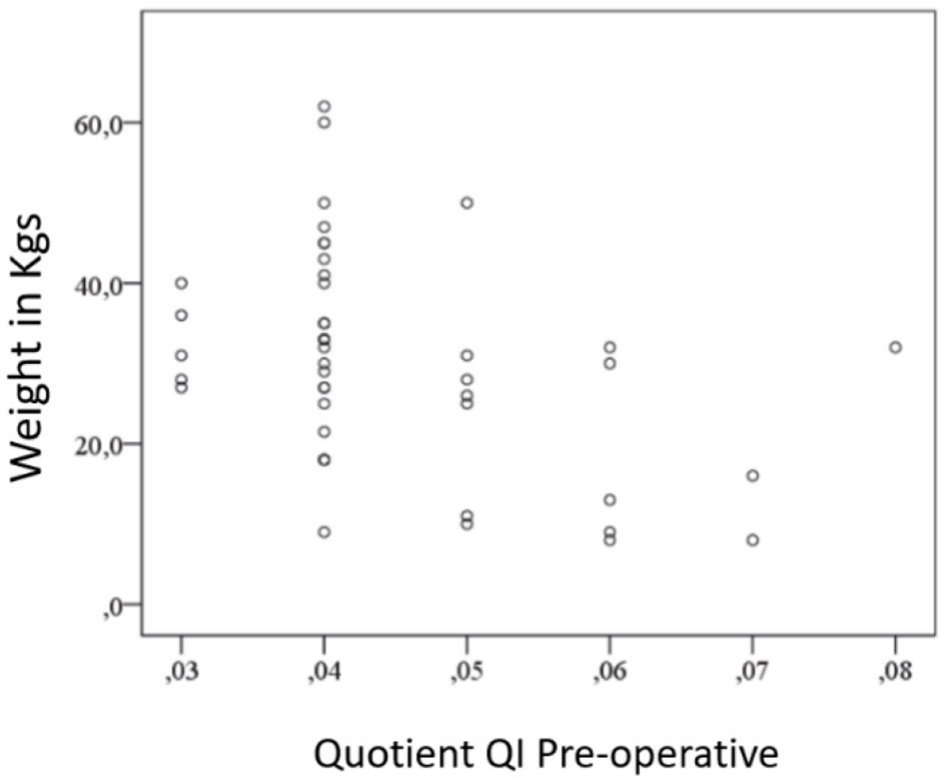
Negative correlation between preoperative QI and body weight.

The negative correlation between body weight and QI preoperatively is illustrated (*r* = −0.307, *p* = 0.008) in Figure 9. This indicates that dogs with a higher body weight tend to have a relatively long or thin patellar ligament.

#### 3.3.3 Cranial drawer test and lameness grade or joint effusion

Regarding the clinical parameters, the drawer test positively correlated with the preoperative lameness grade (*r* = 0.351, *p* = 0.009) and the preoperative joint effusion (*r* = 0.423, *p* = 0.003). The more unstable the stifle joints are, the more pronounced the joint effusion and the greater the lameness in the patient. Additionally, the state of joint effusion 2–3 months post-operatively correlated with the degree of osteoarthritis 2–3 months post-operatively (*r* = 0.382, *p* = 0.007). This relationship was not significant either preoperatively or 8–10 months post-operatively.

#### 3.3.4 Correlation between QI2 and signalment, duration of lameness, surgical findings, and complications

There were no associations between the QI (tendon length and caliber) 2–3 months post-operatively (QI2) and the signalment (age, weight, sex, neuter status), preoperative duration of lameness, surgical findings (partial/full rupture, meniscus damage, joint changes), occurrence of complications, administration of metamizole or NSAIDs, or the performance of professional physiotherapy. No connections were found between the QI2 and clinical parameters (joint effusion, drawer test, hyperextension pain, and muscle atrophy), as well as the degree of osteoarthritis preoperatively and 2–3 months post-operatively.

**Figure 10:**
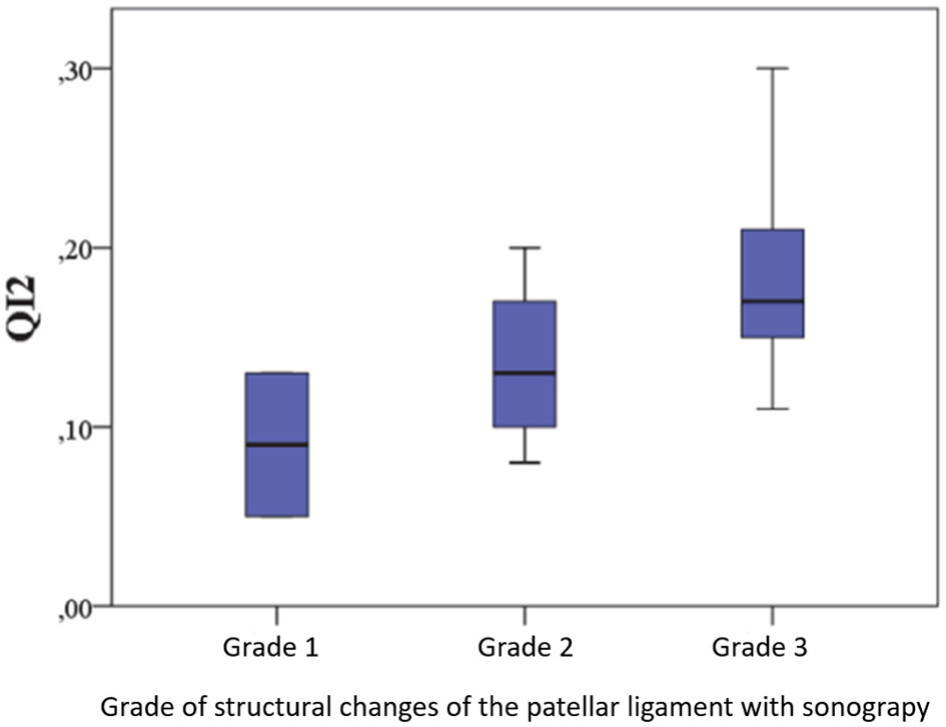
Relationship between QI and structural tendon changes 2–3 months post-operatively.

A significant positive correlation was found between QI2 and ultrasound grading changes 2–3 months post-operatively (*r* = 0.418, *p* = 0.004), indicating that higher-caliber tendons exhibit more pronounced structural changes. This relationship is illustrated in Figure 10.

#### 3.3.5 Correlation between QI3 signalment, duration of lameness, surgical findings, and complications

**Figure 11:**
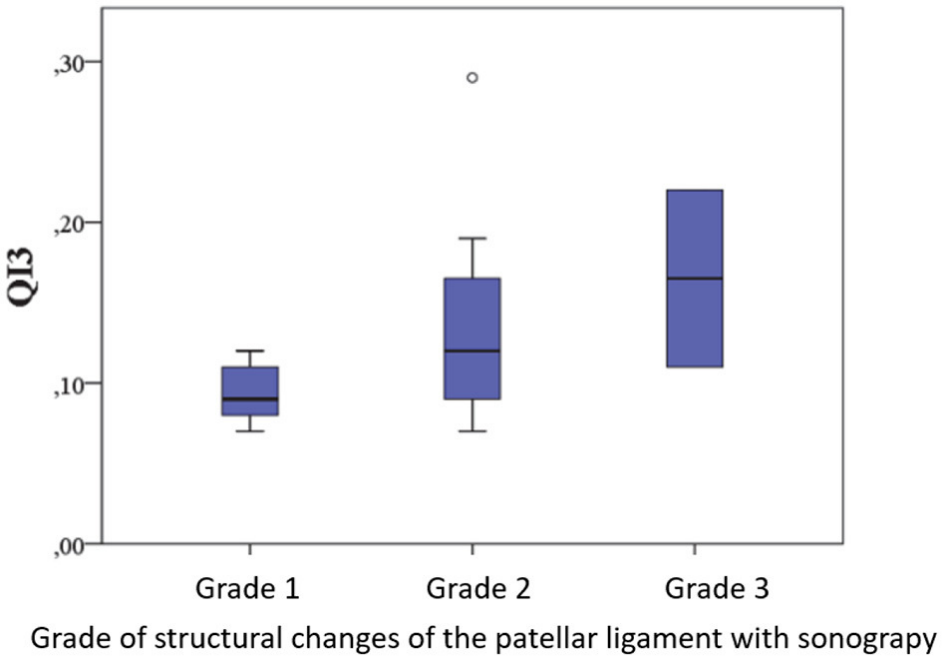
Relationship between QI and structural tendon changes 8-10 months post-operatively.

Similarly, the QI positively correlated with the structural changes 8–10 months post-operatively (QI3) (*r* = 0.400, *p* = 0.009) (Figure 11). Furthermore, no associations were found between QI3 and the accompanying parameters (lameness grade, joint effusion, drawer test, hyperextension pain, muscle atrophy, and osteoarthritis grade). Additionally, these factors had no influence on the difference between QI2 and QI3, nor did the signalment, administration of NSAIDs post-operatively, or physiotherapy.

#### 3.3.6 Correlation between QI and ultrasound grade of structural changes in the patellar ligament

There was a positive correlation between QI2 and QI3 and the ultrasound grades of structural changes at the corresponding postoperative examination time points. The higher the quotients, indicating greater increases in tendon thickness, the more severe the structural tendon changes were. Neither the lameness grade nor the joint effusion, stifle joint stability, hyperextension pain, or osteoarthritis grade influenced the ultrasound structure of the patellar ligament. Similarly, this was independent of the administration of various NSAIDs, the incidence of complications, or the physiotherapy provided.

## 4 Discussion

The rupture of the cranial cruciate ligament (CCL) is the most common cause of lameness in dogs (2–4). While osteotomy techniques aim to alter stifle joint forces (18, 19), the capsular and fascial imbrication (CFI) or Meutstege method (16) achieves stabilization through lateral tightening of the joint capsule. Post-surgical changes in the patellar ligament have been noted, especially following TTAs and TPLOs (7, 10). This study provides the first sonographic analysis of the patellar ligament pre- and post-CFI, assessing correlations with clinical and radiographic findings, as well as factors like medication use, lameness duration, surgical findings, and physiotherapy.

The study included 46 dogs from 17 different breeds. Rottweilers were the most common, with 4 dogs (8.7%), followed by 3 each (6.5%) of Labrador Retrievers and Boxers, and 2 each (4.3%) of Golden Retrievers and German Shepherds. Other purebred breeds and 20 mixed-breed dogs each appeared once in the study. This distribution is generally consistent with earlier research indicating a higher prevalence of cranial cruciate ligament rupture (CCLR) in larger dog breeds (4, 20–24).

The distribution of affected dogs was nearly equal between males (24) and females (22), which aligns with previous findings (25–27). Some studies, however, have reported a higher occurrence in females (28, 29). Large epidemiological studies by Whitehair et al. [(20), covering 602,317 dogs] and Adams et al. [(30), covering 1,368 dogs] also found a higher prevalence in females, although Witsberger et al. (23), in a study of over one million dogs, did not observe this trend. Previous studies have suggested an increased risk of cranial cruciate ligament rupture (CCLR) in neutered dogs (20, 22, 23, 31, 32). However, other studies (30, 33) found no change in risk related to neutering. The exact reasons behind any increased susceptibility remain unclear, though hormonal changes and a tendency for weight gain in neutered dogs (20, 31, 34) are thought to contribute. In terms of sex, Vasseur et al. (35) found no impact on CCL degeneration. Reported ratios of neutered to intact dogs in these studies range widely from 22% to 87.2% (20, 22, 23, 30, 36). In this study, the neutered-to-intact ratio is fairly balanced at 54.3%.

In the present study, patients weighed an average of 29.5 kg, with 67.4% weighing over 25 kg, which aligns with findings from previous research (20, 22, 37). Cranial cruciate ligament rupture (CCLR) is commonly seen in large or overweight dogs, as increased joint stress can accelerate ligament degeneration (4). Additionally, Taylor-Brown et al. (32) observed that, within a breed, heavier individuals are at greater risk, while Guthrie et al. (33) did not find weight to influence CCLR risk.

The average age of patients in our study was 7 years, slightly higher than those reported in other studies, where ages ranged from 2 to 10 years (20, 22, 23, 26, 32). Although our study only included dogs aged one year and older, only 7 (15.2%) were younger than 4 years. This matches epidemiological data from Witsberger et al. (23) and Taylor-Brown et al. (32), which suggest an increased risk of cranial cruciate ligament (CCL) rupture starting at 3–4 years. Whitehair et al. (20) indicated a “risk age” between 7 and 10 years.

In terms of weight and age, we found that 7-year-old dogs averaged 23.9 kg, whereas younger dogs averaged 34.2 kg. Lighter dogs (<10 kg) tended to be older, averaging 11 years, while heavier dogs (>25 kg) averaged 6 years. This pattern is consistent with Bennett et al. (37), and Whitehair et al. (20). Histological studies by Zahm (38) and Vasseur et al. (35), confirm that large-breed dogs experience earlier and more severe CCL degeneration than small-breed dogs. In our findings, age and weight showed a negative correlation. Guthrie et al. (33) observed that CCLR occurs significantly earlier in Rottweilers and later in Golden Retrievers, reflecting breed-specific age patterns.

In our analysis, cruciate ligament ruptures affected the left and right stifle joints with nearly equal frequency, consistent with findings by Bennett et al. (37). However, Guthrie et al. (33) reported that the left stifle joint is more frequently affected, whereas Krotschek et al. (39) found a higher incidence on the right side. Colborne et al. (40) suggest that Labrador Retrievers may favor one side, as treadmill analyses showed an unequal load distribution on their hind limbs.

At the initial presentation, 10 patients already had bilateral CCL ruptures, and in 3 additional cases, the opposite side ruptured during the study. Overall, 13 dogs (28.3%) experienced bilateral CCL ruptures. This aligns with prior studies, where 10–61.3% of dogs initially diagnosed with a CCLR developed a rupture in the opposite stifle within 12–16 months (29, 37, 41–43). Differences in bilateral CCL rupture rates, including delayed ruptures, largely depend on the length of the observation period. For instance, our study's maximum follow-up was 10 months, while Cabrera et al. (43) observed cases for over 2 years, reporting a 61.3% rate. The time from the first rupture to the contralateral tear varies widely, averaging between 32 weeks and 14 months, with longer intervals often observed in older dogs at the time of the initial rupture (36, 37, 43, 44). Younger dogs, particularly young males (44) and Rottweilers (33), are more likely to have a delayed bilateral rupture. However, Buote et al. (36) found no significant correlation between age, weight, or sex and the likelihood of a contralateral rupture.

In our study, 61.2% of cases (30 out of 49) involved a complete rupture of the CCL, which aligns closely with Duval et al. (22), who reported a 66% incidence. Dogs with a complete rupture were, on average, older (8 years) than those with a partial rupture (5.8 years). Additionally, dogs with complete ruptures tended to show more severe joint alterations during surgery, though this difference did not reach statistical significance. Medial meniscus damage occurred in 67.2% of our cases (33 out of 49), which is within the broad range reported in literature, from 33% to 79% (21, 22, 29, 45, 46). The variation in reported rates may be influenced by differences in diagnostic methods—such as clinical exams, X-rays, MRIs, arthrotomy, or arthroscopy—as well as case numbers, symptom duration, and examiner experience.

For our cases, dogs with both a medial meniscus lesion and a CCL rupture had an average weight of 32.1 kg, which was not significantly different from those with an intact meniscus, who averaged 24.7 kg. Consistent with findings by Engelke et al. (46) and Ralphs and Whitney (47), complete CCL ruptures in our study were more frequently associated with meniscal damage than partial ruptures.

In our study, we used a categorical scale to assess the function of the hind limbs. While this scale provides less detail than a continuous visual analog scale, it produces results that are generally comparable (48). Both methods, however, are subjective and lack the sensitivity of the current gold standard, “Force Plate Analysis” (48). At the time of the study, treadmill analysis equipment was unavailable. Preoperative data on the duration of lameness were based on rough estimates provided by dog owners, which led to a simple classification of lameness duration—short (acute, <2 weeks) and long (chronic, more than 2 weeks). However, the duration of lameness did not appear to influence the severity of intraoperative findings (e.g., cruciate ligament damage, meniscus injury, arthritis). A notable observation was that NSAIDs were more frequently administered in cases of long-lasting lameness compared to those with short-term lameness.

At initial presentation, over half of our patients showed moderate to severe lameness. Clinical follow-up at 8 to 10 months post-capsular and fascial imbrication surgery indicated that 77.4% (24 out of 31) of dogs were free of lameness. This outcome aligns with Timmermann's study (25), which reported 76.6% of dogs lameness-free after 6 months, and with Allgöwer et al. (16), who found 93% lameness-free after 3 to 36 months. Weber (49) reported 60% lameness-free after 12 months, whereas Alt (50) found only 47.5% of dogs lameness-free 6 months after surgery using the same technique.

These results do not clarify which factors—such as surgeon experience, patient characteristics, pre- and intraoperative findings, or postoperative rehabilitation—contributed to better or worse functional outcomes. While Ertelt (51) suggested that late functional decline might be due to meniscus damage, Moore and Read (29), Krotschek et al. (39), and our study did not confirm this. Factors such as age, weight, sex, neuter status, meniscus condition, and the preoperative duration of lameness did not influence functional outcomes at any of the three assessment points.

In this sample of patients, stifle joints with a CCL rupture (complete or partial) were palpably more swollen, a finding consistent with Bennett et al. (37). However, Timmermann (25), Duval et al. (22), and Alt (50) did not generally observe this. Post-operatively, the effusion decreased. At 2–3 months post-operation, only 23.5% (8/34) of stifles were completely free of effusion, and at 8–10 months, this percentage decreased to 16.1% (5/31). While these differences were not statistically significant, they are similar to Timmermann's (25) findings, where 19.1% were effusion-free after 6 months. In contrast, Weber (49) reported 54.2% effusion-free after 8 months. These discrepancies can only be speculatively discussed, especially considering that both our study and Weber's (49) study were conducted by the same surgeon. Joint effusion did not correlate with factors such as age, body weight, degree of lameness, pain on hyperextension, muscle atrophy, or NSAID use.

In our study group, 93.5% (43 out of 46) of the stifle joints with a CCL rupture were unstable, showing signs of anterior drawer syndrome. This is consistent with findings from Timmermann (25), Innes and Barr (21), and Alt (50). Two to three months post-surgery, 35.3% (12 out of 34) of the joints were stable, and by 8–10 months, 54.8% (17 out of 31) had become stable.

Eight to ten months post-surgery, the rate of hyperextension pain in our study was 39.7%, higher than Timmermann's (25) rate of 21.3% and Weber's (49) 31.3%. As observed by Weber (49), we also found no correlation between pain during extension and lameness.

Muscle atrophy was visually and palpably assessed in this study but not measured with a tape measure, radiographs (52), or ultrasound (53) as these assessments seemed impractical given the small sample size, including 13 dogs with bilateral CCL ruptures. More than 50% of the dogs show muscle atrophy on the side of the CCL rupture at the initial examination, with the quadriceps femoris muscle being particularly affected (52). Interestingly, no significant link was found between preoperative lameness duration and muscle atrophy, possibly due to 32 dogs having received preoperative NSAIDs. Muscle atrophy increased significantly in the first 2–3 months post-surgery, likely due to inactivity. However, by the second follow-up 8–10 months post-surgery, muscle mass had returned to preoperative levels in all but two dogs, who still exhibited notable atrophy.

Although many studies strongly recommend starting physiotherapy soon after surgery for effective rehabilitation (21, 54–56), our findings do not support this approach, which aligns with the results of Jerre (57). It should be noted that neither Jerre (57) nor our study recommended targeted physiotherapy; rather, we simply advised gradually increasing the animal's activity on a leash. This “do-it-yourself” physiotherapy recommendation led to a trend toward functional improvement through muscle growth at the 2–3 and 8–10 month follow-ups, with *p*-values of 0.014 and 0.015, respectively. However, professional physiotherapy was performed in only 11 (29.7%) cases in our study, which limits any conclusions about its impact on muscle mass gain or clinical outcomes. Further studies are needed to clarify this aspect.

Only one dog had stifle and hip joints free from osteoarthritis pre-surgery, similar to previous findings (25). A 25% incidence of unaffected joints was reported by Brunnberg (17). Gonarthrosis in CCL-affected joints ranges from 50.2% to 91.4% (16, 17, 25, 50, 58), with large-breed and overweight dogs generally considered at higher risk (59, 60), which our results support. No correlation was identified between lameness duration and preoperative osteoarthritis, aligning with previous studies (37), though others observed such a relationship (21). Preoperatively, 30.4% (14/46) had mild (grades 1–3), 39.1% (18/46) moderate (grades 4–6), and 23.9% (11/46) severe (grades 7–9) gonarthrosis/gonotrochlosis. Osteophytes were first observed 21–60 days after experimentally cutting the CCL in dogs (59, 61). In our study, 61.1% (11/18) of dogs with acute lameness (CCL rupture <14 days old) had moderate to severe osteoarthritis, though the causes remain speculative.

During follow-ups, operated joints showed gonarthrosis/gonotrochlosis, with 44.1% (15/34) severely deformed 2–3 months post-surgery and 64.5% (20/31) after 8–10 months. Only one small dog (<10 kg) exhibited mild osteoarthritis. The progression of gonarthrosis in our study aligns with findings from Tirgari (62), Vasseur and Berry (63), Rayward et al. (64), and Boyd et al. (65). Despite differences in surgical techniques, such as intra- vs. extra-articular methods (45), and comparisons between TTA and TPLO (13, 66), all methods show varying degrees of gonarthrosis progression. TPLO-treated joints are less affected by osteoarthritis than CFI-treated joints, but still show progression: 40% and 76% with mild osteoarthritis in 2.5% and 21% of cases, respectively (64, 65).

In our study, at the first follow-up (2–3 months post-surgery), the extent of joint effusion positively correlated with the degree of osteoarthritis. These findings support Goldenberg et al. (67) and Johnson (68), suggesting that osteoarthritis progression and synovitis influence each other through joint instability, without impacting lameness severity (13, 50, 60, 65, 69, 70).

In a group of 46 dogs, we conducted a comparative ultrasound exam just before surgery, under anesthesia, using an 11 MHz transducer. For 20 dogs, the unaffected stifle joint served as a control. Preoperatively, the patellar ligament appeared smooth, homogenous, and slightly hyperechoic, with a fibrillar texture in the longitudinal view and a granular, oval shape in cross-section—consistent with previous studies (11, 14, 15, 71). No significant differences were observed between CCL-affected and healthy stifles. In 15.2% (7/46) of cases, the ligament showed mild thickening (Grade 1), differing from rates reported by others (5, 11, 72). The reason for these variations remains unclear.

In our group, the sonographically measured average ligament thickness on the CCL-ruptured side was 1.6 mm, compared to 1.5 mm on the healthy side. This aligns closely with previous findings, which reported measurements of 1.8, 1.7, and 2.1 mm in the middle of the patellar ligament on the CCL-affected side (5, 7, 11). Compared to radiographic measurements reported by others, including values of 2.6, 3.22, 2.68, and 3.77 mm (5, 7, 8, 73), ultrasound proves more sensitive and accurate for measuring ligament thickness, as it better delineates the ligament from surrounding tissue (5, 7, 11).

In our study, structural changes in the patellar ligaments, assessed via ultrasound, were classified. The average ligament thickness increased significantly post-operatively to 5.4 mm, exceeding measurements reported in other studies. In contrast, previous studies found smaller increases after similar surgeries: Mattern et al. (11) documented postoperative thicknesses following TPLO at 2.7 mm one month post-op, 2.5 mm after 2 months, and 2.4 mm after 6 months. For TTA procedures, Kühn et al. (5) and Pettitt et al. (7) reported thicknesses of 2.9 and 2.57 mm 6 weeks post-TTA and 2.9 mm and 1.9 mm at later checkups (16 weeks and 6 months post-op, respectively). However, radiographic studies, such as those by Carey et al. (10) and Owen et al. (73), reported much larger thicknesses, exceeding 12 and 11.6 mm.

In our control group, the average ligament thickness at the initial postoperative check remained consistent with preoperative measurements (1.5 mm). Eight to ten months post-surgery, 41.9% of CCL-affected patellar ligaments still showed moderate to severe changes, while 58.1% were slightly thickened but structurally intact, indicating significant improvement. Comparatively, Mattern et al. (11) found 38.5% of ligaments remained moderately to severely altered 6 months after TPLO, and Kühn et al. (5) reported 18.8% structural changes 16 weeks post-TTA. Over time, our study also showed a significant decrease in ligament thickness, reaching an average of 3.9 mm, paralleling findings from Mattern et al. (11) and Pettitt et al. (7), though they did not assess statistical significance. DeSandre-Robinson et al. (8) reported a significant reduction in ligament thickness between four and eight weeks post-TTA, whereas no decrease was noted post-TPLO at three and six weeks; similarly, Kühn et al. (5) observed no significant changes between six and 16 weeks post-TTA.

Significant structural and thickness differences were observed between the healthy and operated joints. Notably, none of the ligaments in the affected joints returned to preoperative thickness, whereas prior studies report regression rates of 38.5% and 6.3% (5, 11).

Thickening of the patellar ligament after surgical treatment of CCL ruptures has been frequently reported, as summarized in supplementary Table 1. However, differences in study populations, surgical and examination methods, and follow-up times make direct comparisons challenging. Only three studies used ultrasound to examine ligament changes, with most relying on radiography. No studies were found assessing patellar ligament changes after capsular and fascial imbrication (CFI), although Berger (13) noted radiographic thickening after it, with no such changes seen in dogs under 15 kg after TPLO. Nevertheless, various studies (8–11, 73, 74) documented thickened patellar ligaments post-operatively.

In this study, ultrasound was chosen to assess changes in patellar ligament thickness and structure, as it is considered superior to X-ray, MRI, and CT for this purpose (5, 75). However, ultrasound's accuracy is examiner-dependent, which can introduce errors (76). The literature cites various factors that can lead to ligament thickening and structural changes after cruciate ligament rupture, independent of surgical technique, often attributed to biomechanical alterations (5, 7–11, 73).

Unlike previous studies, our findings did not attribute increased ligament thickness to the overlapping fascia reinforcement, as proposed by Berger (13); instead, we clearly identified separate structures like the patellar ligament, fascia, and connective tissue. Structural changes are likely due to the suture technique, causing inflammatory responses and temporary thickening post-surgery (77, 78). Further studies could explore if alternative suture materials, such as nylon, result in less ligament reaction. Limitations of the study include a modest sample size, loss of patient follow-up during various check-ups due to owner non-compliance, and a relatively short follow-up period of only 10 months. Additionally, some limbs experienced contralateral rupture, which prevented their use as controls in subsequent examinations.

Post-operative thickening of the patellar ligament appears to be a consequence of various cranial cruciate ligament rupture repair methods. However, its exact cause and implications remain unclear. This study provides insight into this common condition, suggesting that ligament thickening does not necessarily correlate with clinical outcomes. Our study found that patient age, sex, body weight, cruciate ligament, or meniscal condition did not influence postoperative ligament thickening or structural changes. This aligns with previous findings (5, 6, 73) but contradicts reports by Mattern et al. (11) and Carey et al. (10). Similarly, ligament changes showed no correlation with lameness severity, joint effusion, drawer test results, hyperextension pain, or muscle atrophy, consistent with human studies (79–83). This study is the first to sonographically document temporary structural and thickness changes in the patellar ligament following CFI for CCL rupture, although these changes had no impact on clinical or radiological outcomes. Future studies could explore histological correlations to further characterize these findings. The capsular and fascial imbrication technique has proven effective and cost-efficient surgical treatment for CCLR (16, 84), and these results provide insights into the ligament's structural response to this surgical method and its clinical relevance compared to other CCLR surgical treatments.

## 5 Conclusions

This prospective study provides valuable insights into the sonographic changes in the patellar ligament following capsular fascial imbrication surgery for cranial cruciate ligament (CCL) rupture in dogs. Ultrasonographic evaluation demonstrated significant postoperative increases in both the thickness and structural alterations of the patellar ligament. These changes were most pronounced at 2–3 months post-surgery, with a notable decrease in structural alterations by 8–10 months, although the ligament never fully returned to its preoperative state.

The study found that postoperative patellar ligament thickness correlated positively with structural changes, indicating that greater increases in thickness were associated with more severe alterations. However, no significant correlation was observed between tendon changes and clinical or radiological findings, including lameness severity, joint effusion, or the presence of arthritis. Furthermore, no associations were identified between tendon changes and preoperative factors such as the duration of lameness, NSAID use, or other intraoperative findings.

The lack of significant differences in tendon changes across different patient characteristics (such as age, weight, and sex) further underscores the complexity of the post-surgical rehabilitation process. While no direct clinical disadvantages were associated with the observed sonographic changes, the results contribute to understanding the structural dynamics of the patellar ligament post-surgery.

## Data Availability

The original contributions presented in the study are included in the article/Supplementary material, further inquiries can be directed to the corresponding author.
