# Supplementary material for: Ultrasonographic examination of the patellar ligament after capsular and fascial imbrication for the treatment of cranial cruciate ligament rupture in dogs
Source: Front Vet Sci. 2025 Mar 25;12:1544445. doi: 10.3389/fvets.2025.1544445 (PMC11977418; doi:10.3389/fvets.2025.1544445)
Supplement: Supplementary file 1 [file Table_1.docx]

Supplementary Material

Table 1: Literature references on patellar tendinopathies following surgical treatment of cranial cruciate ligament rupture (TPLO: tibial plateau leveling osteotomy, TTA: tibial tuberosity advancement, CFI: capsular-fascial imbrication)

| Author (Year) | Nr. of Cases | Aver-age age | Average weight | Percentage of tendinopathi-es | Method | Post operative observa-tion period | Examina-tion method |
| --- | --- | --- | --- | --- | --- | --- | --- |
| PACCHIANA et al. (2003) | 397 | 5 years | 39.9 kg | 14.3% | TPLO | - | X-ray |
| CAREY et al. (2005) | 94 | 5 years | 36.8 kg | 79.8%* | TPLO | 2nd month | X-ray |
| STAUFFER et al. (2006) | 696 | 6.2 years | 38.4 kg | 2.7% | TPLO | up to 30 months | X-ray |
| MATTERN et al. (2006) | 31 | 5.2 years | 33.4 kg | 100.0% | TPLO | 1st, 2nd, & 6th month | X-ray + Ultrasound |
| KÜHN et al. (2011) | 21 | 7 years | 26.7 kg | 100.0% | TTA | 6th & 16th week | X-ray + Ultrasound |
| STEHLÍK et al. (2013) | 20 | - | - | - | TTA | 7th & 15th week | X-ray |
| BERGER (2014) | 21 | 7.0 years | 9.88 kg | 0.0% | TPLO | ≥ 6 months | X-ray |
|  | 17 | 9.9 years | 9.25 kg | 100.0% | CFI | ≥ 6 months | X-ray |
| COLETTI et al. (2014) | 1519 | 5.4 years | 37.3 kg | 0.3% | TPLO | up to 6 months | X-ray |
| PETTITT et al. (2014) | 19 | 4.7 years | 36.8 kg | 72.0% | TTA | 6th week & 6th month | X-ray + Ultrasound |
| BARNES et al. (2016) | 29 | 7 years | 10.3 kg | 58.3% | TPLO | up to 26 weeks | X-ray |
| DESANDRE-ROBINSON et al. (2017) | 59 | 4.4 years | 33.1 kg | 97.0% | TPLO | 3rd & 6th week | X-ray |
|  | 47 | 5.6 years | 33.0 kg | 92.0% | TTA | 4th & 8th week | X-ray |
| OWEN et al. (2018) | 199 | 5.4 years | 33.4 kg | 78.0%* | TPLO | 8-12 weeks | X-ray |


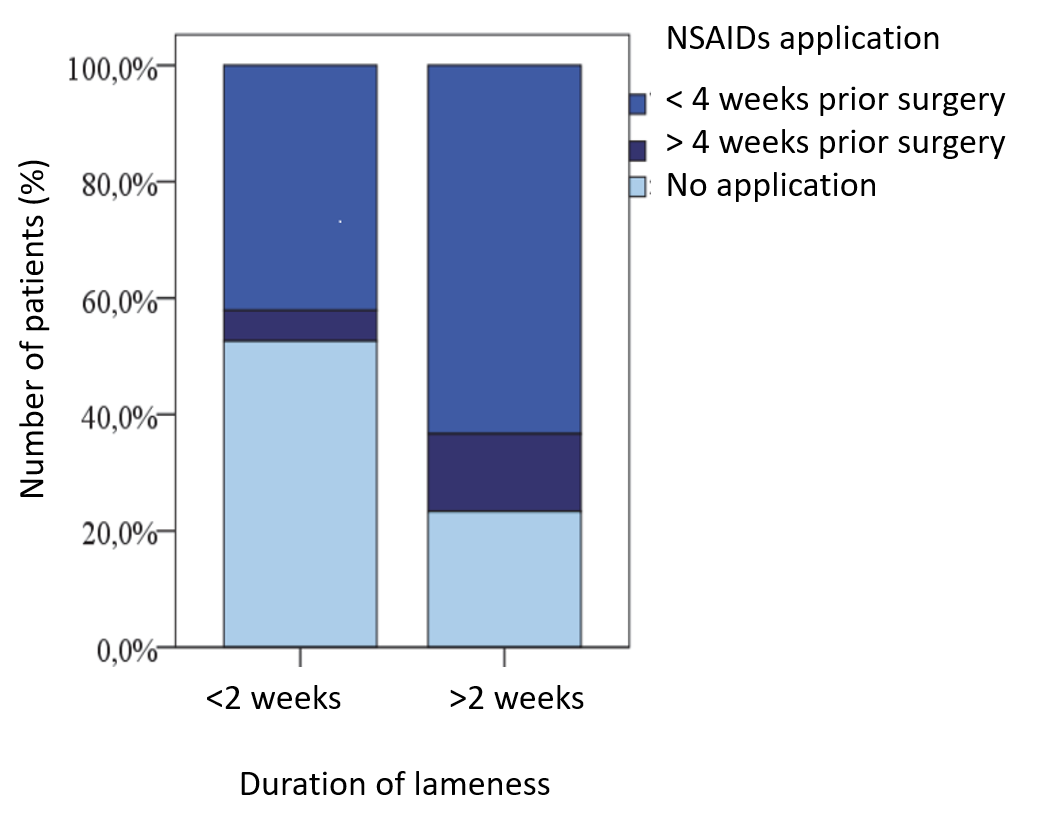


Figure 1: Relationship between preoperative NSAID application and duration of lameness


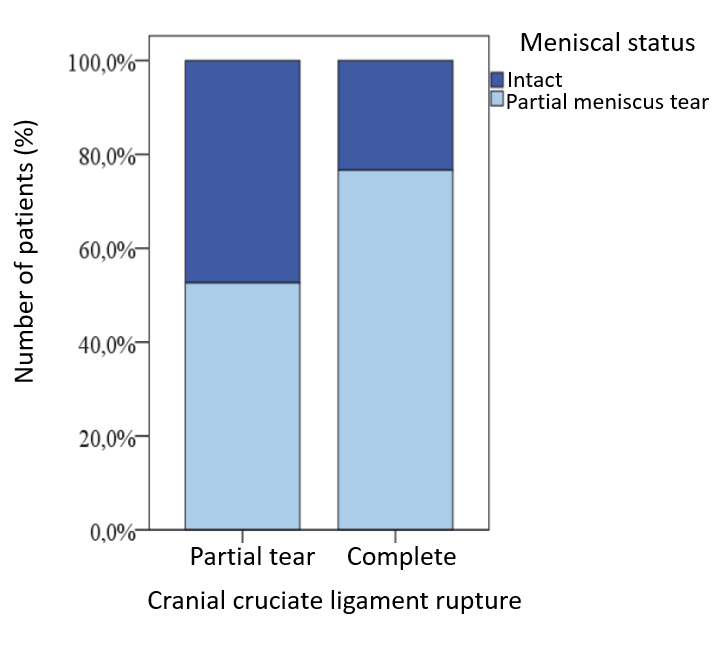


Figure 2: Meniscal findings in partial versus complete cruciate ligament rupture


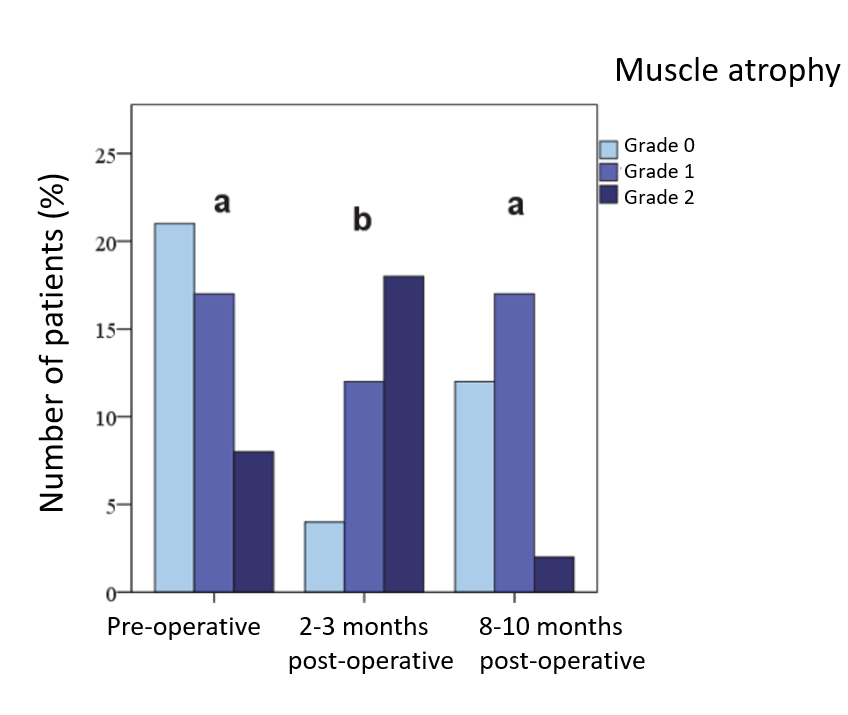


Figure 3: Severity of muscle atrophy before and at various time points after surgery


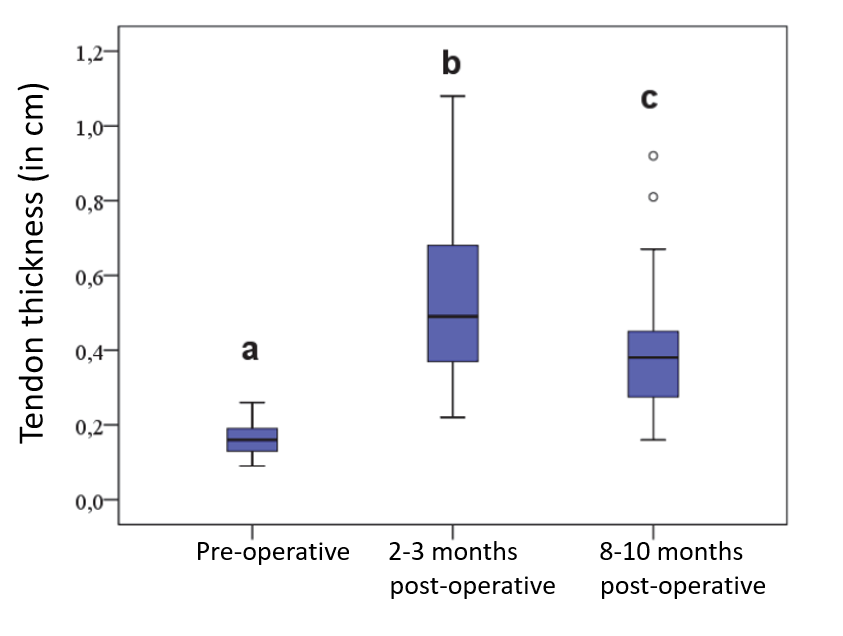


Figure 4: Caliber of the patellar ligament before and at various time points after surgery


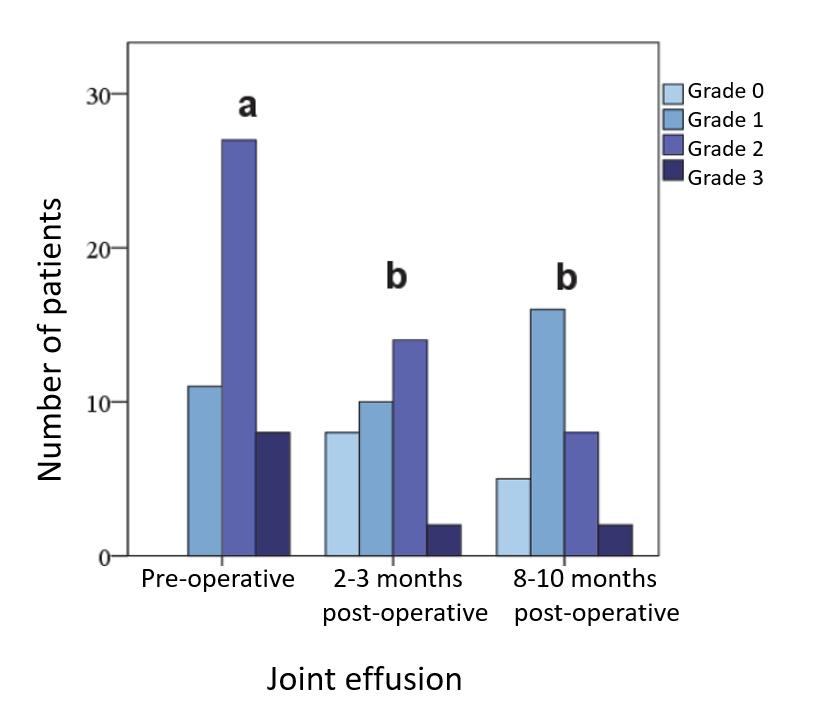


Figure 5: Joint effusion before and at various time points after surgery


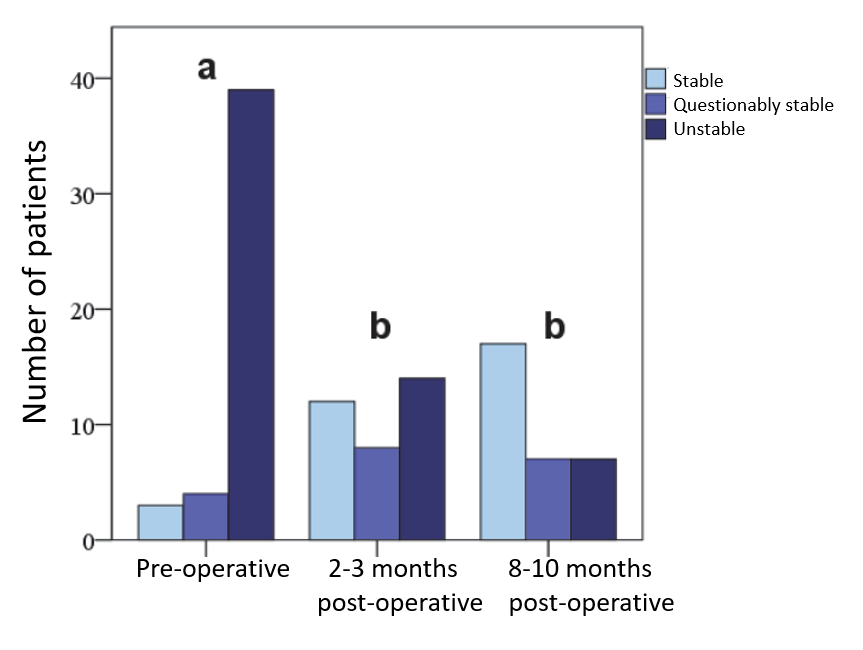


Figure 6: Results of the cranial drawer test before and at various examination time points after the operation
